# The Danish health and morbidity surveys: study design and participant characteristics

**DOI:** 10.1186/s12874-019-0733-9

**Published:** 2019-05-03

**Authors:** Heidi Amalie Rosendahl Jensen, Ola Ekholm, Michael Davidsen, Anne Illemann Christensen

**Affiliations:** National Institute of Public Health, University of Southern Denmark, Copenhagen, Denmark

**Keywords:** Cross-sectional studies, Study design, Data collection, Follow-up studies, Health surveys, Health surveillance

## Abstract

**Background:**

Reliable data from health surveys are essential to describe the status and trends in health indicators by means of information not available from official registers. In Denmark, nationally representative health surveys (the Danish Health and Morbidity Surveys) have been carried out among adults during the past three decades by the Danish National Institute of Public Health, University of Southern Denmark. The aim of the present study is to describe the study design of the three most recent surveys in 2010, 2013, and 2017, including the survey mode and response rates.

**Methods:**

In 2010, 2013, and 2017, the samples (*n* = 25,000 each) were based on random sampling of individuals aged 16 years or older with a permanent residence in Denmark. A subsample of previously invited respondents was also re-invited in subsequent survey waves. Data were collected through self-administered questionnaires, yet with a concurrent mixed-mode approach, allowing for the invited individuals to complete either a web questionnaire or an identical paper questionnaire. In 2010 and 2013, survey invitations were sent by regular postal mail, whereas a secure electronical mail service, Digital Post, was used to invite the majority (90.1%) of the sample in 2017.

**Results:**

The overall response rate decreased from 60.7% in 2010 to 57.1% in 2013 and 56.1% in 2017. Between 2010 and 2017 the response mode distribution for the web questionnaire increased markedly from 31.7 to 73.8%. The largest increase in the proportion which completed the web questionnaire was found in the oldest age group.

**Conclusions:**

Data from the Danish Health and Morbidity Surveys reveal an increasing proportion of the respondents to complete web questionnaires instead of paper questionnaires. Even though the response rate remained relatively stable in 2017, declining response rates is a major concern in health surveys. As the generalizability to the Danish population may be compromised by a low response rate, efforts to increase the response rate or keep it stable are crucial in future surveys. Thus, efforts should be made to ensure convenience and feasibility in relation to access to and the completion of survey (web) questionnaires.

## Background

Comprehensive public health surveillance systems are crucial in health care planning and policy development [1, 2]. To obtain such systems, health surveys constitute an essential component. As information collected in health surveys typically covers other topics than do official statistical registers, e.g. indicators of self-rated physical and mental health, health behavior (e.g. alcohol consumption, smoking, and physical activity), and quality and quantity of social relations, data from health surveys provide a unique opportunity to generate a more diverse, yet precise, picture of a population’s health status and trends. Moreover, register data on health care contacts provide only information on the most serious medical conditions, for which medical treatment was necessary (e.g. acute myocardial infarction, stroke, and cancer). This means that data on conditions that are generally considered less serious than those previously mentioned, albeit more common in the daily life of the general population, are not included in registers (e.g. allergy, headache, and osteoarthritis) [1]. Thus, such information can only be revealed by means of health surveys. Moreover, in many countries, adequate official health registers are not available, and, accordingly, health surveys are the only source of data on the population’s health.

There are also some potential disadvantages of using health survey data as a proxy of the general population’s health. Firstly, a tendency towards declining response rates in surveys has been observed in recent years in several countries [3, 4]. If the non-response rate is high and if non-respondents deviate markedly from respondents, the representativeness and generalizability of the survey data are compromised [2]. However, if non-response is missing at random, such non-response bias does not limit the validity of survey data [4, 5]. Another potential limitation of survey data validity is the risk of recall bias [6] and bias related to social desirability, i.e. the tendency of respondents to over-report ‘healthy behaviours’ and underreport ‘unhealthy behaviours’ [7].

Besides monitoring health by means of data derived from official statistical registers, Denmark has a long tradition of monitoring health in the general population though surveys. In Denmark, it is also possible to link on responses from surveys on an individual level with comprehensive and precise registry data on e.g. hospitalisations, drug prescriptions, and mortality, made feasible because each individual has a unique personal identification number. Since 1987, the Danish National Institute of Public Health, University of Southern Denmark, has regularly carried out nationally representative health surveys. Data from these surveys have been widely used in both national and international monitoring of health and morbidity indicators, e.g. by Eurostat, the World Health Organization (WHO), the Organisation for Economic Co-operation and Development (OECD), and the European Monitoring Centre for Drugs and Drug Addiction (EMCDDA). Also, data from these surveys have been used in several publications in international peer-reviewed scientific journals (e.g. [8–11]).

The purpose of the present paper is to describe the study design, including the mode of data collection, response rates and samples of three most recent waves of the Danish Health and Morbidity Surveys, which were carried out in 2010, 2013, and 2017.

## Methods

The Danish Health and Morbidity Surveys have been carried out in 1987, 1994, 2000, 2005, 2010, 2013, and 2017 [12–17]. The overall aim of the surveys is to describe the status and trends in health and morbidity in the adult Danish population aged 16 years or older. Moreover, the aim is to describe the factors that may influence the population’s health status, including e.g. health behaviour, mental health, and environmental health risks.

### Sample design

In Denmark, each individual has a unique personal identification number. This allowed for all survey samples to be drawn at random from the adult population using the Danish Civil Registration System [18]. The register contains information on matters such as sex, age, address, marital status, citizenship, and place of birth among all individuals with a permanent residence in Denmark [18].

The sample design of the surveys in 1987, 1994, 2000, and 2005 is described in detail elsewhere [19, 20]. Since 2010, the Danish Health and Morbidity Survey has been incorporated into the Danish National Health Survey, which is based on six mutually exclusive random subsamples, one from each of the five Danish regions and one national sample, the latter being the Danish Health and Morbidity Survey [21]. Along with this incorporation, the mode of data collection in the Danish Health and Morbidity Survey was changed, too. Compared to questions included in the questionnaire from the Danish National Health Surveys, more question on sensitive matters such as illicit drug use, gambling, sexual health, and sexual assaults are included in the questionnaires from the Danish Health and Morbidity Surveys.

In 2010, the survey was based on a study sample of 25,000 individuals aged 16 years or older with a permanent residence in Denmark and was constituted by two sub-samples: 1) a follow-up sample of individuals invited to participate in earlier survey waves (*n* = 6142) and 2) a supplementary sample ensuring a nationally representative sample size of 25,000 individuals (*n* = 18,858) in total. The follow-up sample was constituted by: a) individuals invited to participate in the survey in 1994 along with supplementary samples in 2000 and 2005 (*n* = 5322), b) a random sample of individuals who were between 16 and 20 years old and had a permanent residence in Denmark in 2010 (*n* = 460), and c) a random sample of non-Danish citizens (*n* = 360). Hence, the follow-up sample of 6142 individuals was constructed to be nationally representative of the adult population aged 16 years or older in Denmark in 2010. The supplementary sample (*n* = 18,858) was also drawn at random to reflect the adult population in Denmark.

In 2013, the same sampling approach was used as in 2010, resulting in a study sample of 25,000 individuals aged 16 years or older with a permanent residence in Denmark. The two sub-samples were: 1) a follow-up sample of individuals invited to participate in earlier survey waves (*n* = 5517) and 2) a supplementary sample ensuring a nationally representative sample size of 25,000 individuals (*n* = 19,483) in total. The follow-up sample was constituted by: a) individuals invited to participate in the survey in 1994 along with supplementary samples in 2000, 2005, and 2010 (*n* = 5232) and b) a random sample of individuals who were between 16 and 18 years old and had a permanent residence in Denmark in 2013 (*n* = 285). The total follow-up sample of 5517 individuals was nationally representative of the adult population in Denmark in 2013. The supplementary sample of 19,483 individuals was randomly selected among the adult population in Denmark aged 16 years or older.

In 2017, a similar sampling approach was applied to that used in 2010 and 2013. Thus, the total study sample included 25,000 randomly selected individuals aged 16 years or older who had a permanent residence in Denmark in 2017. The two sub-samples were: 1) a follow-up sample constituted by individuals invited to participate in earlier survey waves (*n* = 5150) and 2) a supplementary sample that ensured that the sample size was nationally representative and included a total of 25,000 individuals (*n* = 19,850). The follow-up sample was constituted by: a) individuals invited to participate in the survey in 1994 along with supplementary samples in 2000, 2005, 2010, and 2013 (*n* = 4800) and b) a random sample of individuals between age 16 and 19 years with a permanent residence in Denmark (*n* = 350). In total, the follow-up sample of 5150 individuals was nationally representative of the adult population in Denmark in 2017 (16 years or older). The supplementary sample of 19,850 individuals was also drawn at random to reflect the adult population in Denmark.

### Data collection

Initially, an introduction letter was sent to all selected individuals that briefly described the purpose and content of the survey. It was emphasized that participation was voluntary. In 2010 and 2013, the introduction letters were sent by postal service, but in 2017 it was decided to distribute the introduction letters digitally by the secure electronical mail service, Digital Post. As a rule, all individuals in Denmark are registered to use Digital Post; however, a smaller proportion of the study population (9.9%) had actively deregistered from the service. This group of individuals, primarily constituted by elderly, was sent an introduction letter by regular postal service.

In 2010, 2013, and 2017, a concurrent mixed-mode approach was used to collect the survey data, allowing for the invited individuals to complete either a web questionnaire or to fill out an identical enclosed paper questionnaire. For each individual, the introduction letter contained a unique user name and password that enabled access to the web questionnaire. In the Central Denmark Region sample it was, however, only possible to complete a paper questionnaire in 2010. In 2017, a slightly different concurrent mixed-mode approach than in 2010 and 2013 was used to collect the survey data: Initially, all selected individuals registered to use Digital Post (90.1% of the sample; the proportion decreased from 98.7% among individuals aged 16–24 years to 68.7% among individuals aged ≥65 years) were electronically invited to complete only the web questionnaire. In contrast, individuals who were not registered to use Digital Post were invited by letter to complete the web questionnaire or an identical paper questionnaire. Thus, the sample in 2017 was constituted by these two subsamples.

In 2010, 2013, and 2017, reminders were sent to all invited individuals who had not yet completed and returned or submitted the questionnaire, excluding those who had actively indicated that they did not want to participate in the survey. However, the numbers of reminders varied across the survey waves as well as within the two subsamples in 2017. In 2010 and 2013, a total of two reminders, excluding the introduction letter, were sent by letter to the individuals who had not already completed and returned or submitted either the paper questionnaire or the web questionnaire. Enclosed in both the introduction letter and the second reminder were a paper questionnaire and a pre-paid return envelope. In 2017, a total of four reminders, excluding the introduction letter, were sent to the subsample that was initially invited through Digital Post. If the web questionnaire was not completed after 1 week, an electronic reminder was sent to non-response individuals. After yet another 3 weeks of non-response in the Digital Post subsample, these individuals were approached by a reminder letter sent by regular postal service. Enclosed in this letter was a paper questionnaire identical to the web questionnaire and a pre-paid return envelope. The remaining two reminders to the initial Digital Post subsample were sent by regular postal service, the last one with an enclosed paper questionnaire and a pre-paid return envelope. In contrast, individuals who had initially been invited by regular postal service to complete the paper or web questionnaire received only two reminders, excluding the introduction letter. In the introduction letter as well as in the last reminder there was an enclosed paper questionnaire and a pre-paid return envelope.

### Weighting

In all three survey waves, weights were constructed by using auxiliary information form Statistics Denmark’s registers in order to take into account the different sampling probabilities. As all individuals with a permanent residence in Denmark have a unique personal identification number, it was possible to link on an individual level the personal identification numbers of both respondents and non-respondents to relevant central registers. Hence, by applying calibrated weights it was to some extent possible to statistically allow for the differential non-response. Weights were computed by Statistics Denmark and based on information on e.g. sex, age, municipality of residence, highest completed level of education, income, marital status, ethnic background, number of visits to the general practitioner 3 years prior to each survey wave, occupational status, and owner/tenant status. Statistics Denmark was responsible for the construction of weights only.

Descriptive statistics (i.e. percentages) were used to present the results. Furthermore, descriptive statistics were also used to describe the characteristics of the follow-up sample (e.g. the number of invited individuals and respondents, respectively).

## Results

In 2010, 2013, and 2017, the questionnaire was fully or partially completed by 15,165, 14,265, and 14,022 respondents, respectively (Table 1). As the samples in each of the three survey waves were comprised by a total of 25,000 individuals, the overall response rate thus declined from 60.7% in 2010 to 57.1% in 2013 and 56.1% in 2017. In each survey year, the response rate was lower among men (2010: 56.4%; 2013: 52.9%; 2017: 51.6%) than among women (2010: 64.9%; 2013: 61.1%; 2017: 60.6%) and particularly low among men aged 16–24 years (2010: 41.5%; 2013: 40.8%; 2017: 39.6%) and 25–44 years (2010: 49.0%; 2013: 43.9%; 2017: 40.8%). Moreover, the response rate varied according to marital status and ethnic background. Accordingly, there was a low response rate among unmarried individuals and among individuals with an ethnic background other than Danish.

**Table 1:** Response rate according to sex, age, marital status, and ethnic background among the sample. Percentages

	Response rate		
	2010	2013	2017
Total	60.1	57.1	56.1
Men			
16–24y	41.5	40.8	39.6
25–44y	49.0	43.9	40.8
45–64y	61.7	57.8	57.1
≥65y	69.3	65.2	66.3
All men	56.4	52.9	51.6
Women			
16–24y	58.5	54.1	52.7
25–44y	62.4	57.9	55.0
45–64y	70.2	66.7	66.6
≥65y	63.8	60.7	63.3
All women	64.9	61.1	60.6
Marital status			
Married	68.5	64.6	64.4
Divorced	56.8	53.8	54.8
Widowed	56.6	53.1	55.0
Unmarried	50.3	47.6	46.2
Ethnic bacground			
Danish	63.3	59.8	58.9
Western	42.1	40.5	40.7
Non-Western	33.7	29.4	33.2
Sample size	25,000	25,000	25,000
No. of respondents	15,165	14,265	14,022

According to Table 2, there was a clear tendency for respondents to be more likely to complete the web questionnaire over time. Thus, the overall proportion of respondents completing the web questionnaire increased from 31.7% in 2010, to 41.5% in 2013 and 73.8% in 2017. There was an increase among both men and women and in all age groups; however, the increase was most pronounced among men and women in the older age groups. For example, the proportion of respondents who completed the web questionnaire increased from 13.4% in 2010 to 67.2% in 2017 among men aged 65 years or older and from 5.9% in 2010 to 60.1% in 2017 among women aged 65 years or older.

**Table 2:** Data mode distribution (web) according to sex, age, marital status, and ethnic background among the sample. Percentages

	Web questionnaire		
	2010	2013	2017
Total	31.7	41.5	73.8
Men			
16–24y	51.5	65.7	84.8
25–44y	53.8	60.9	77.9
45–64y	38.5	48.7	78.6
≥65y	13.4	22.3	67.2
All men	38.0	46.4	76.0
Women			
16–24y	36.6	52.9	75.0
25–44y	37.0	49.9	73.0
45–64y	27.6	40.3	79.0
≥65y	5.9	14.4	60.1
All women	26.3	37.5	71.9
Marital status			
Married	31.1	40.3	75.2
Divorced	28.7	36.1	71.5
Widowed	7.8	13.1	52.2
Unmarried	40.1	52.6	76.5
Ethnic bacground			
Danish	31.4	41.1	73.6
Western	33.9	48.2	74.5
Non-Western	38.5	48.8	76.1

Table 3 shows the characteristics of the follow-up samples (the 2010–2013 follow-up sample, the 2010–2017 follow-up sample, and the 2013–2017 follow-up sample). The follow-up baseline sample in 2010 was constituted by a total of 6142 individuals, 2550 of whom completed a self-administered questionnaire in the survey in both 2010 and 2013, and 2306 respondents completed a self-administered questionnaire in both 2010 and 2017. In 2013, the total baseline sample consisted of 5517 individuals. A total of 2923 participants completed a self-administered questionnaire in both 2013 and 2017. Altogether, 2001 participants completed a self-administered questionnaire in all three survey years (2010, 2013, and 2017).

**Table 3:** Overview of the follow- up samples. Number of individuals (Percentages)

2010–2013	Baseline sample	Respondents in 2010	Invited in 2013	Follow-up study population
Total	6142	3762	5232	2550
Sex				
Men	3049 (49.6)	1722 (45.8)	2585 (49.4)	1152 (45.2)
Women	3093 (50.4)	2040 (54.2)	2647 (50.6)	1398 (54.8)
Age				
16–24y	837 (13.6)	432 (11.5)	766 (14.6)	258 (10.1)
25–44y	1986 (32.3)	1116 (29.7)	1584 (30.3)	688 (27.0)
45–64y	2102 (34.2)	1388 (36.9)	1893 (36.2)	1042 (40.9)
65 + y	1217 (19.8)	826 (22.0)	989 (18.9)	562 (22.0)
2010–2017	Baseline sample	Respondents in 2010	Invited in 2017	Follow-up study population
Total	6142	3762	4875	2306
Sex				
Men	3049 (49.6)	1722 (45.8)	2411 (49.5)	1027 (44.5)
Women	3093 (50.4)	2040 (54.2)	2464 (50.5)	1279 (55.5)
Age				
16–24y	837 (13.6)	432 (11.5)	720 (14.8)	219 (9.5)
25–44y	1986 (32.3)	1116 (29.7)	1535 (31.5)	626 (27.2)
45–64y	2102 (34.2)	1388 (36.9)	1818 (37.3)	1008 (43.7)
65 + y	1217 (19.8)	826 (22.0)	802 (16.5)	453 (19.6)
2013–2017	Baseline sample	Respondents in 2013	Invited in 2017	Follow-up study population
Total	5517	3147	5150	2297
Sex				
Men	2730 (49.5)	1439 (45.7)	2552 (49.6)	1040 (45.3)
Women	2787 (50.5)	1708 (54.3)	2598 (50.4)	1257 (54.7)
Age				
16–24y	840 (15.2)	368 (11.7)	798 (15.5)	217 (9.5)
25–44y	1503 (27.2)	779 (24.8)	1447 (28.1)	561 (24.4)
45–64y	1895 (34.3)	1178 (37.4)	1842 (35.8)	937 (40.8)
65 + y	1279 (23.2)	822 (26.1)	1063 (20.6)	582 (25.3)

## Discussion

The Danish Health and Morbidity Surveys are nationally representative surveys that have been carried out regularly for 30 years and currently comprise a total of seven survey waves. For some health indicators it is therefore possible to monitor trends across the whole period. However, different modes of data collection were applied until 2010, and it has been shown that the data collection mode affects prevalence estimates for some indicators, potentially introducing bias if indicators were compared across all seven survey waves [22]. Since 2010, the mode of data collection has been harmonised in the Danish Health and Morbidity Surveys, which makes it possible to monitor various trends in all health indicators over time in the three most recent survey waves (2010, 2013, and 2017). This is essential in relation to e.g. health surveillance, planning and prioritising public health initiatives and research.

In the present study, the overall response rate was 56.1% in 2017. Response rate comparisons to other surveys are difficult as differing definitions of response rates is a well-recognised issue [23]. By applying a uniform definition of both numerator and denominator Mindell et al. [23], however, compared response rates across surveys in seven European countries carried out between 2007 and 2017 among individuals aged between 25 and 64 years. Albeit no overall country-specific response rates were reported in the study, a similar pattern in relation to age and sex was found as that demonstrated in the present study, i.e. the response rate was higher among women than men and increased with increasing age. The observed low response rate among young individuals, especially among men aged 16–24 years, is, of course, a matter of concern in the present study. However, the use of calibrated weights made it possible to statistically adjust for the differential non-response e.g. among young men. This means that the responses among e.g. men in age group 16–24 years were given a certain weight so that their impact had a higher weight to account for the low response rate overall within this group. Hence, the use of calibrated weights is, to our knowledge, the best way to minimize the impact of low response rates in certain age groups in a population survey.

In the present study, the observed decline in the response rate between 2010 (60.7%) and 2013 (57.1%) leveled off and remained broadly stable in 2017 (56.1%). A possible explanation that the response rate did not decline further in 2017, despite overall declining response rates in several countries in the past decades [3, 4], is the slightly different concurrent mixed-mode approach applied in the 2017 survey wave. This approach implied that all selected individuals registered to use Digital Post (90.1% of the sample) were initially electronically invited to complete only a web questionnaire. The remaining 9.9% of the sample were invited by regular postal service to complete the web questionnaire or an identical paper questionnaire. In Denmark, Digital Post is typically used by public authorities, e.g. health authorities, and private companies such as banks and insurance companies to contact a specific citizen and deliver a message to the person concerned. Electronic mails sent by Digital Post are sent encrypted, which means that the digital security is very high and higher than for mails sent by regular postal service and e-mails. Because spam is a large and ubiquitous part of the Internet, successful administration of web surveys is essential in order to make the respondents not treat legitimate survey contact e-mails as spam [24]. The use of Digital Post in the survey in 2017 may therefore have resolved this issue, and it seems likely that respondents registered to use Digital Post may have perceived the introduction letter and thus the survey itself as more serious than in earlier survey waves where the introduction letters were sent by regular postal service [23]. Moreover, as public authorities such as municipalities and hospitals use Digital Post to inform citizens about e.g. medical examinations, it is possible that respondents who are regularly contacted by such authorities, including citizens who are typically underrepresented in health surveys, are more likely to receive, read and react to the survey invitation than if contacted by regular postal service.

In addition to the rather formal and serious built-in value contained in e-mails sent by Digital Post, there are several other general benefits of using web questionnaires in health surveys [25–27]. Firstly, it is possible for the respondents to complete the web questionnaire on the go and in steps. This is convenient for many people and thus makes survey participation feasible. Moreover, the software in web questionnaires facilitates automatic branching, i.e. skipping of unnecessary or non-applicable questions [28]. For researchers, the benefits of using web questionnaires in surveys include faster and cheaper data collection [26]. Despite the fact that initial costs for web surveys vary depending on the level of programming sophistication required, the cost per response declines as the number of respondents increases, as data entry is performed by the respondents themselves [29]. This offers potential savings compared to postal or telephone survey modes where material and staff costs tend to be proportional to respondent numbers [29].

Another possible explanation for the leveling off in 2017 of the previously observed decline in the response rate may relate to the number of reminders sent to the respondents registered to use Digital Post. Excluding the introduction letter, a total of four reminders were sent to respondents in this subsample. In contrast, only two reminders were sent to respondents not registered to use Digital Post in 2017 (as well as to all respondents in 2010 and 2013). Previous research has shown that a higher number of reminders sent to respondents in surveys increase the response rate [30, 31]. Thus, as the response mode distribution for web questionnaire steadily increased from 31.7% in 2010 to 73.8% in 2017 and 90.1% of the respondents were registered to use Digital Post in 2017, which implied receiving four reminders, these factors are likely to counterbalance the general tendency of declining response rates observed in previous studies [3, 4].

It cannot be ruled out that some of the invited individuals who were contacted by regular postal service did not receive (and open) these mails, which then may have affected the response rate and the response mode distribution. However, during the data collection the postal service used to deliver the mails made random telephone calls to assess the success rate of the postal mail delivery, which confirmed a high success rate. We do as well not know if all individuals invited by Digital Post opened this mail, as we did not have access to such data. Further, the combined use of both postal mail and digital post has most likely increased the chance that the majority of the invited individuals has received the survey invitation, including the questionnaire.

The major strengths of the Danish Health and Morbidity Surveys are that 1) they are large nationally representative studies, which have been carried out regularly for three decades, allowing for the monitoring of trends in various health indicators over time 2) data derived from the surveys can be linked on an individual level to different official statistical registers (e.g. the Danish National Patient Register, the Danish Register of Causes of Death, The Danish National Prescription Register, and the Danish National Service Register) due to the unique personal registration numbers, which allows for analyses of the relationship between e.g. risk factors and morbidity and mortality, social inequality in health etc., 3) the questionnaires cover a wide variety of topics not included in official statistical registers.

Declining response rates in surveys is a major concern, as the generalizability of the collected data to the target population may be compromised if the non-response rate is high [2, 32]. Another concern in this regard is when both the response rate and the characteristics of non-respondents change over time. Accordingly, it may be difficult to determine whether the observed changes in estimates are real or whether they are merely due to changes in response rates and in the representativeness of the results [3]. To account for these challenges, information on non-respondents was obtained from official statistical registers in the present surveys, allowing us to carry out non-response analyses and, thus, to a certain extent statistically adjust for differential non-response by applying calibrated weights. Another general limitation of survey data is the cross-sectional design, which does not allow conclusions to be drawn on the direction of causality [32]. However, because of the study design in the Danish Health and Morbidity Surveys, which includes follow-up samples, data can be used for both cross-sectional and longitudinal analyses. Accordingly, it is also possible to study causal relationships between e.g. health behavior or other risk factors and morbidity and health, as well as to carry out follow-up analyses in official statistical registers.

The mixed-mode design of the present surveys also introduces some potential issues of concern as it may lead to internal measurement errors due to mode effects [21, 25]. Mode effects occur when the content or outcome of data obtained from one mode of data collection differs from that obtained from another. When applying a mixed-mode approach in data collection, it is therefore important to consider the potential impact of the mode of data collection on the data [21]. However, several evaluations have shown no significant mode effects across different target populations and topics [31, 33–36], and a large body of research suggests that the benefits associated with a mixed-mode approach outweigh the potential challenges [25–27]. Moreover, the potential mode effects in the present surveys are probably relatively small, as both modes - i.e. filling in a web or a paper questionnaire, respectively - include the self-administration of a questionnaire.

Finally, the reliability of self-reported survey data is based on confidence in the accuracy of the respondents’ recall as well as on their motivation to provide truthful information on the topic of interest. However, when examining conditions such as hypertension or diabetes in population surveys, one should keep in mind that such diagnoses formally also require physical examinations and biomarker data to be given. Therefore, self-reported survey data on such and similar conditions could result in underestimated prevalence rates.

## Conclusion

The Danish Health and Morbidity Surveys are nationally representative health surveys that have been carried out regularly since 1987. Since 2010, the data collection method has been harmonized, thus allowing for direct comparisons of the included health indicators over time. In the present study, we demonstrated that the declining trend in the response rate between 2010 and 2013 leveled off in 2017. As the data mode distribution between 2010 and 2017 showed an increasing proportion of the respondents to complete web questionnaires instead of paper questionnaires, this may to some extent explain why the response rate did not further decline in 2017. Moreover, in 2017 the introduction letter was distributed to the majority of the sample by the secure electronical mail service, Digital Post, which may have influenced the invited individuals’ perception of the survey. Lastly, a total of four reminders were sent to the subsample registered to use Digital post, which may have positively affected the response rate or at least stagnated the overall declining trend. When feasible, future surveys are encouraged to take into account the demonstrated increasing preference for completing web questionnaires in surveys in order to increase or remain stable the response rate. However, this may not be possible in all countries, e.g. in countries with a high degree of heterogeneity or without a secure electronical mail service (as Digital Post in Denmark).
